# Supplementary material for: Geomapping Vitamin D Status in a Large City and Surrounding Population—Exploring the Impact of Location and Demographics
Source: Nutrients. 2020 Aug 31;12(9):2663. doi: 10.3390/nu12092663 (PMC7551618; doi:10.3390/nu12092663)
Supplement: Supplementary file 1 [file nutrients-12-02663-s001.pdf]

Supplementary Materials

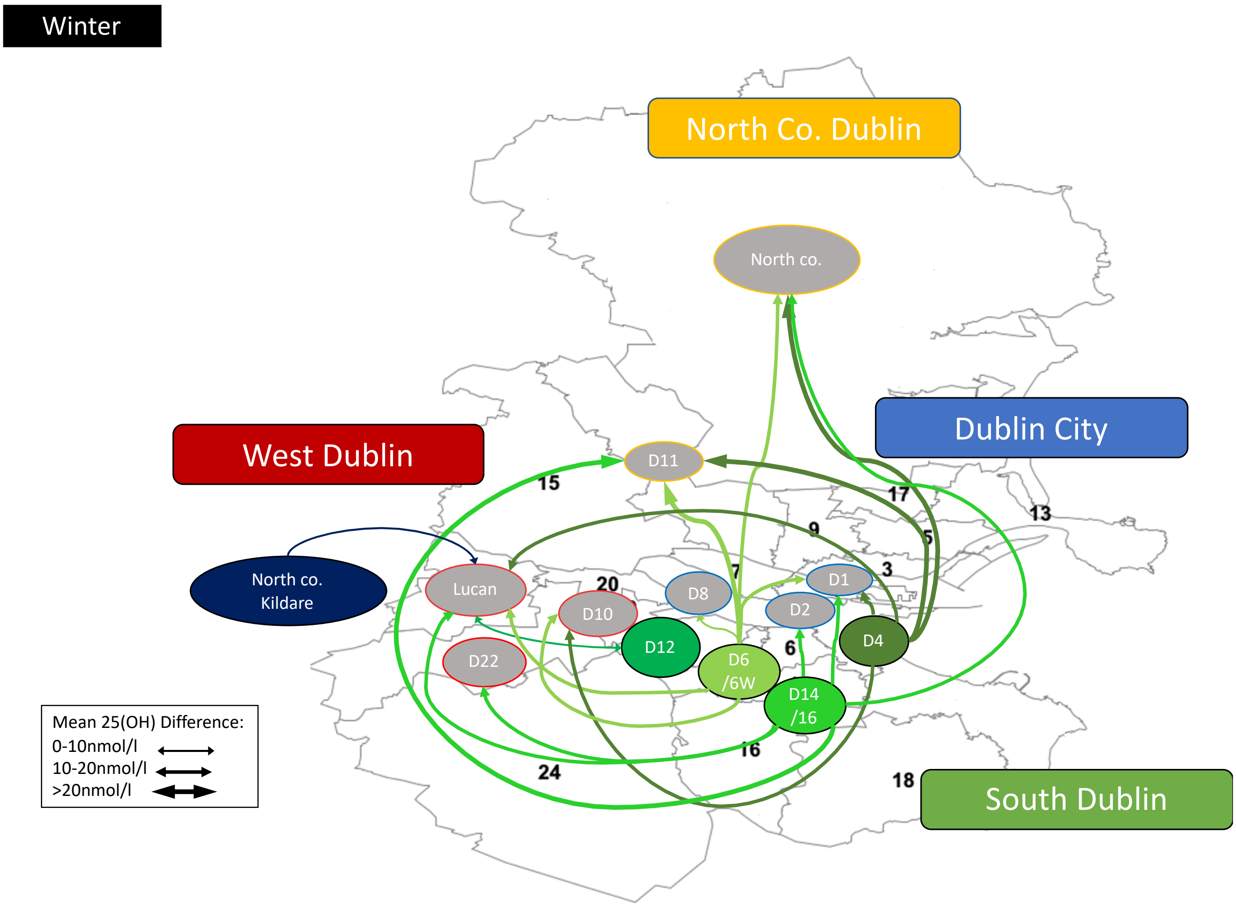

Figure S1. Cluster map analysis in winter. Areas with significant mean differences illustrated using coloured arrows for each area (going in the direction of higher to lower status) and with arrow size representing the size of this difference.

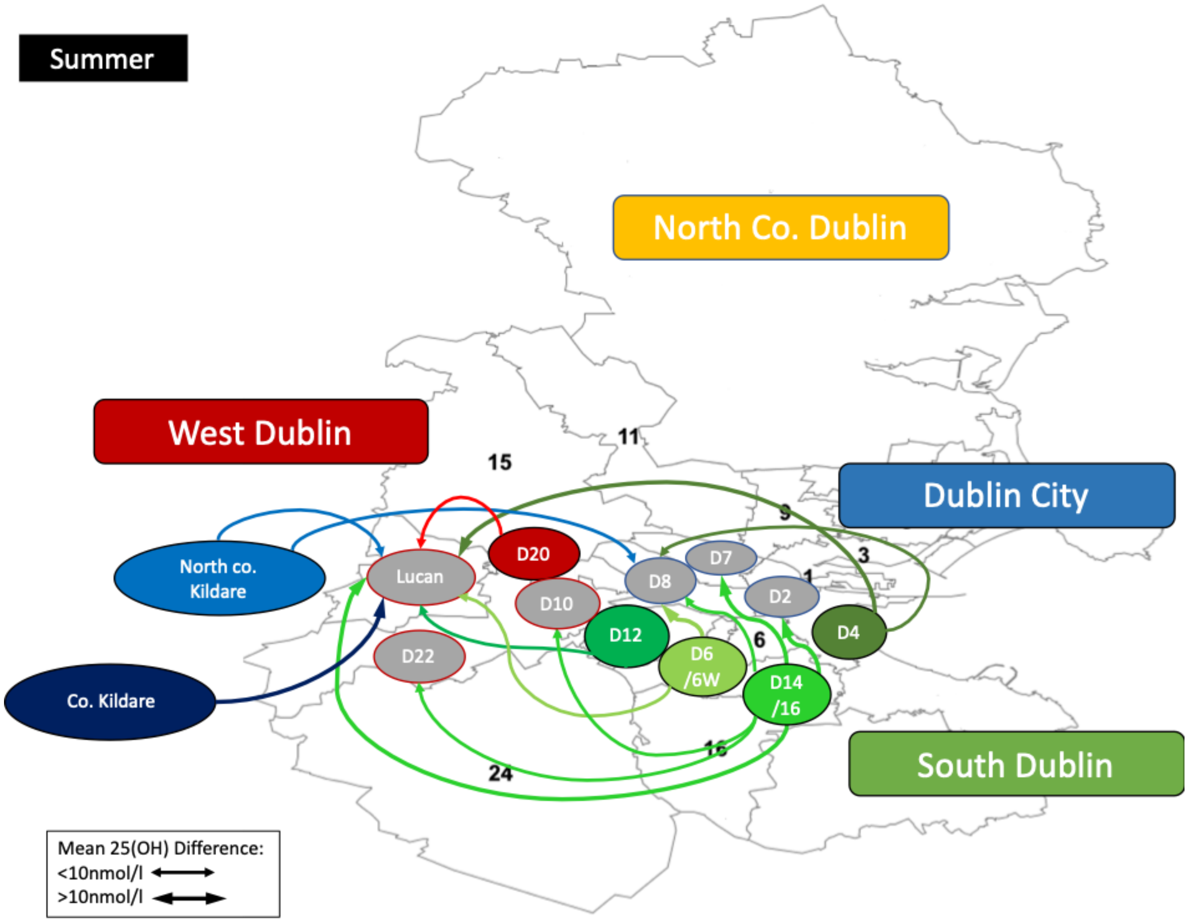

Figure S2. Cluster map analysis in Summer.

Table S1. Condensed Pairwise ANOVA Winter. All mean difference is significant at the <0.05 level.

	Dublin 1	Dublin 2	Dublin 8	Dublin 10	Dublin 11	Dublin 22	North Co. Dublin	Lucan
**Dublin 4**	19.3 (4.3)			15.0 (3.5)	25.9 (5.8)		21.7 (5.2)	14.0 (2.8)
**Dublin 6/6W**	15.6 (3.5)		6.5 (1.5)	11.4 (2.5)	22.3 (5.3)		18.2 (4.6)	10.3 (1.3)
**Dublin 12**								7.3 (1.4)
**Dublin 14/16**	17.2 (3.6)	10.8 (2.6)	8.0 (1.3)	12.9 (2.5)	23.8 (5.3)	11.7 (2.8)	19.7 (4.6)	11.9 (1.3)
**North Co. Kildare**								7.4 (1.2)

Table S2. Condensed Pairwise ANOVA Summer. All mean difference is significant at the 0.05 level.

	Dublin 2	Dublin 7	Dublin 8	Dublin 10	Dublin 22	Lucan, Co. Dublin
**Dublin 4**			9.8 (2.5)			13.2 (2.4)
**Dublin 6/6W**			6.1 (1.4)			9.5 (1.2)
**Dublin 12**						9.0 (1.3)
**Dublin 14/16**	12.0 (2.6)	11.9 (2.6)	10.0 (1.4)	8.14 (2.1)	9.4 (2.3)	13.5 (1.3)
**Dublin 20**						8.6 (1.8)
**North Co. Kildare**			6.4 (1.4)			9.8 (1.2)
**Rest of Kildare**						10.9 (2.4)
